# Construction of a QSAR Model Based on Flavonoids and Screening of Natural Pancreatic Lipase Inhibitors

**DOI:** 10.3390/nu15153489

**Published:** 2023-08-07

**Authors:** Yutong Yuan, Fei Pan, Zehui Zhu, Zichen Yang, Ou Wang, Qing Li, Liang Zhao, Lei Zhao

**Affiliations:** 1 Beijing Engineering and Technology Research Center of Food Additives, School of Food and Health, Beijing Technology and Business University, Beijing 100048, China; s20223061183@cau.edu.cn (Y.Y.); zhuzehui0614@163.com (Z.Z.); zcyang@st.btbu.edu.cn (Z.Y.); 2002020217@st.btbu.edu.cn (Q.L.); 2 Beijing Key Laboratory of Functional Food from Plant Resources, College of Food Science and Nutritional Engineering, China Agricultural University, Beijing 100083, China; 3 Institute of Apicultural Research, Chinese Academy of Agricultural Sciences, Beijing 100093, China; yunitcon@yeah.net; 4 National Institute for Nutrition and Health, Chinese Center for Disease Control and Prevention, Beijing 100050, China; wangou@ninh.chinacdc.cn

**Keywords:** pancreatic lipase, dietary flavonoids, quantitative structure-activity relationship, molecular simulation, molecular mechanics/generalized born surface area

## Abstract

Pancreatic lipase (PL) is a key hydrolase in lipid metabolism. Inhibition of PL activity can intervene in obesity, a global sub-health disease. The natural product is considered a good alternative to chemically synthesized drugs due to its advantages, such as low side effects. However, traditional experimental screening methods are labor-intensive and cost-consuming, and there is an urgent need to develop high-throughput screening methods for the discovery of anti-PL natural products. In this study, a high-throughput virtual screening process for anti-PL natural products is provided. Firstly, a predictable anti-PL natural product QSAR model (R^2^_train_ = 0.9444, R^2^_test_ = 0.8962) were developed using the artificial intelligence drug design software MolAIcal based on genetic algorithms and their conformational relationships. 1068 highly similar (FS > 0.8) natural products were rapidly enriched based on the structure-activity similarity principle, combined with the QSAR model and the ADMET model, for rapid prediction of a total of five potentially efficient anti-PL natural products (IC_50pre_ < 2 μM). Subsequently, molecular docking, molecular dynamics simulation, and MMGBSA free energy calculation were performed to not only reveal the interaction of candidate novel natural products with the amino acid residues of PL but also to validate the stability of these novel natural compounds bound to PL. In conclusion, this study greatly simplifies the screening and discovery of anti-PL natural products and accelerates the development of novel anti-obesity functional foods.

## 1. Introduction

The increasing prevalence of obesity and its associated complications is becoming a growing public health problem [1]. Obesity is a significant contributor to the chance of developing numerous chronic diseases, including cancer, diabetes, hypertension, hyperlipemia, and cardiovascular disease [2]. Pancreatic lipase (PL) inhibitors play a key role in the metabolism of human fat. It breaks down the oil in the food source into small molecules of glycerol and fatty acids so that the body can absorb these substances and synthesize new fats through lipid metabolism [3]. Based on this feature, the search and development of PL inhibitors can control lipolysis at the source and thus intervene in obesity. Orlistat is a potent, specific, and irreversible inhibitor of pancreatic and gastric lipase, which exerts its pharmacological activity by forming covalent bonds with the active serine sites of gastric and PL in the lumen of the gastrointestinal (GI) tract [4]. Therefore, there is an urgent need to find safe and effective anti-obesity therapies and drugs to address these problems. 

Natural products have been applied in anticancer [5], metabolic regulation [6], and antiviral [7] research due to their structural variety, low toxicity, and wide range of sources. Flavonoids are a rich and representative branch of natural products that are widely found in natural plants with polyphenolic structures [8]. Research reports that foods rich in flavonoids have good anti-obesity effects. Zhang et al. reported that *C. tinctoria* and its flavonoid kaempferol showed protective effects against diet-induced disorders of glucose metabolism and intestinal microbial changes in obese mice [9]. Hao et al. reported that flavonoids in seed residues of hippophae rhamnoides have inhibitory effects on obesity [10]. In order to clarify the relationship between flavonoids and PL, Li et al. determined the effects of 26 kinds of dietary flavonoids on PL inhibitory activity and discovered that flavonoids exhibited stronger PL inhibitory activity [11]. Li et al. employed various spectroscopic techniques and computational simulations to study the inhibitory effect of apigenin, a natural dietary flavonoid, on PL activity and found that apigenin could reversibly inhibit PL activity in a competitive mode [12]. These results suggest that flavonoids are effective PL inhibitors as well as functional anti-obesity factors. However, flavonoids with different structural features, such as different basic units, polymer size, and degree of glycosylation, showed different PL inhibitory effects [13]. Therefore, exploring the intrinsic link between the molecular structures of different flavonoids and their PL inhibitory activity and discovering novel, highly active food-derived anti-PL active compounds could help prevent the risk of obesity as well as reduce the number of obese people.

In recent years, quantitative structure–activity relationship (QSAR) models have been widely used for the quantitative analysis of the structure–activity relationships of compounds, such as the effects of flavonoids on Alzheimer’s disease, oxidative metabolism, and influenza virus inhibition [14,15,16]. However, QSAR studies on flavonoid and PL inhibitory activity are rarely reported. It is of great significance to automatically screen out suitable natural analog candidate inhibitors based on rapid matching of the prediction model. In this study, QSAR models were established based on previous experimental data [13,17]. The 1444 2D molecular descriptors of compounds in the dataset were derived from PaDEL-Descriptor, and feature filtering and QSAR model construction were performed using MolAICal’s GA algorithm for predicting PL inhibitory activity of flavonoids [18]. In this study, we developed a comprehensive computational strategy for the rapid screening of flavonoids as natural PL inhibitors. Based on this QSAR model, combined with Molnatsim, 1068 structurally similar substances were obtained in the COCONUT natural product cluster library. Five PL inhibitors with the lowest IC_50_ values were further screened by ADMET and molecular docking [19]. Finally, molecular dynamics (MD) simulations and molecular mechanics/generalized born surface area (MMGBSA) energy calculations were performed to validate the potential pharmacokinetics of these candidate inhibitors and their binding stability to PL. These results contribute to the search for effective PL inhibitors and provide an accurate model for the further development of novel PL inhibitor derivatives. 

## 2. Materials and Methods

### 2.1. Dataset

A total of 40 flavonoids were selected from flavones, flavanones, isoflavones, flavonols, and flavanols (Table S1), all of which had an obvious inhibitory effect on PL. We set the IC_50_ concentrations in µM and calculated the pIC_50_ value according to the formula −log IC_50_ × 10^−6^. The pIC_50_ value was used as the dependent variable in the QASR modeling analysis [19]. The data set was randomly split into two sets: a training set (70%) and a test set (30%), taking structural variety and bioactivity range into consideration. After creating a classification model with 29 compounds from the training set, 11 compounds from the test set were used as additional independent samples to verify the reliability and stability of the classification model.

### 2.2. Molecular Descriptors

The flavonoids in the dataset were searched in the PubChem database [1], https://pubchem.ncbi.nlm.nih.gov/ (accessed on 26 November 2022), and the SDF format of their 3D structures was obtained. To remove undesired atomic contacts and geometries, the 3D structures of all flavonoids were optimized using the Avogadro program [20], and energy was minimized using the steepest descent method of the MMFF94s force field [21].

Descriptors can convert chemical information encoded in the molecular symbol representation into a useful number or some standard experimental result [22]. SDFs of compounds in the dataset were imported into the PaDEL-Descriptor software package to calculate molecular descriptors (version 2.21, http://www.yapcwsoft.com/dd/padeldescriptor/ (accessed on 3 August 2022)) [23]. PaDEL descriptors contain 1875 descriptors (1444 1D and 2D descriptors and 431 3D descriptors). The 2D molecular descriptors represent structural information that can be calculated from the 2D structure of the molecule, such as the number of benzene rings, the number of hydrogen bond donors, etc. The 3D molecular descriptors represent structural details that can only be learned from the molecule’s 3D representation (e.g., the solvent-accessible and surface area of the structure with a positive partial charge).

The descriptors were preprocessed before modeling to improve the stability and sensitivity of the model. To eliminate high covariance parameters (*p* > 0.9), correlation analysis was used on the calculated molecular descriptors. 

### 2.3. Model Construction and Prediction of pIC_50_ Values

QSAR is a powerful computational method for analyzing data based on chemical structure. In order to predict biological activity with a variety of target chemical products, the QSAR pharmacophore model was developed by creating a statistical mathematical connection between calculated chemical descriptors of molecular structure and experimentally measured values of these molecules’ biological activity [24].

In this study, 2D-QSAR models were built by calculating the molecular properties of the training and test sets using MolAICal. Multiple linear regression (MLR) methods were used to build 2D-QSAR models. Five descriptors were used as independent variables as well as pIC_50_ as the dependent variable to establish statistical linear correlations for the training set data (29 compounds) [25]. 

Finally, the descriptors were further optimized to construct the QSAR-MLR equations based on the GA algorithm. These parameters and the pIC_50_ of flavonoids were optimized iteratively using genetic algorithms separately, and QSAR models were constructed in which 70% of dipeptide compounds were used as training sets and the remaining 30% as validation sets. The optimal QSAR model was selected on the basis of three validation parameters (R^2^, Q^2^, and MAE) [26]. All five descriptors incorporated into the model are characterized in Table 1.

eATSC6e, centered Broto-Moreau autocorrelation-lag6/weighted by Sanderson electronegativities; GATS2p, geary autocorrelation-lag2/weighted by polarizabilities; SpMin8_Bhi, smallest absolute eigenvalue of Burden modified matrix-n8/weighted by relative first ionization potential; VR2_D, normalized Randic-like eigenvector-based index from Barysz matrix/weighted by atomic number.

### 2.4. Validation of Models and Selection of the Optimal One for Prediction

The parameters R^2^ fitting, adjusted R^2^, MAE, RSS, and SDEC were used to assess the MLR-QSAR model’s predictability and stability. The description and calculation formula of these parameters are as follows:

R^2^ fitting: Correlation coefficients between experimental and predicted values, which were calculated using Equation (1):

(1) $$R^{2}=1-\frac{{SS}_{res}}{{SS}_{tot}}=1-\frac{{\sum_{i}(y_{i}-f_{i})}^{2}}{{\sum_{i}(y_{i}-\bar{y})}^{2}}$$

R^2^ adjusted: The adjusted R^2^ is defined as Equation (2):

(2) $$\mathrm{adjusted}\;R^{2}=1-\frac{PRESS∕df_{e}}{{SS}_{tot}∕df_{t}}$$

MAE: Mean absolute error was calculated using Equation (3):

(3) $$\mathrm{MAE}=\frac{1}{m}\sum_{i=0}^{m}|y_{i}\;-\;f_{i}|$$

RSS: Residual sum of squares was calculated using Equation (4):

(4) $$\mathrm{RSS}=\sum_{i}{\left(y_{i}-f_{i}\right)}^{2}$$

SDEC: Standard deviation error in calculation was calculated using Equation (5):

(5) $$\mathrm{SDEC}=\sqrt{\frac{RSS}{n}}$$

### 2.5. Natural Product Screening Based on the MCS Algorithm and ADMET

The MolNatSim tool based on the COCONUT database was used to match the similarity of each flavonoid in this study for potential PL inhibitors. The MolNatsim tool was built on a pre-optimized molecular similarity prediction model (molecular ECFP4 fingerprint and Mini Batch K-means algorithm [27]). Finally, the generated MLR-QSAR models and ADMET prediction analysis were used to assess their potential as natural PL inhibitors. The PreADMET online program website (https://preadmet.bmdrc.kr (accessed on 12 March 2023)) was used to assess the water solubility, bioavailability Score, GI absorption, P-glecoprotein inhibition in vitro, percutaneous permeability coefficient log Kp (cm/s), and carcinogenicity in rats and mice.

### 2.6. Molecular Docking

Molecular docking was performed using the AutoDock Vina code (Version 1.1.2). The structure of human PL (PDB code: 1LPB) was obtained from the RCSB Protein Data Bank library. To prepare the receptor molecule, the 1LPB was preprocessed in PyMOL by splitting the A and B chains, using AutoDock 4.0 to fix deletions and terminal residues, removing water molecules, assigning atom types, and inserting hydrogen atoms. The SDF files of the small molecules were downloaded from PubChem online, and the MMFF94s force field in Avogadro was used to minimize the energy of the small molecules. The rotatable bonds of the ligands were detected and assigned using AutoDocktool. The position of the docking box was referenced from published literature and adjusted slightly [28].

In the whole docking process, semiflexible docking is adopted, and the target protein PL remained rigid at all times while all kinks of the inhibitor were free to rotate. The grid coordinates used for molecular docking research are as follows: PL (x = 11.282, y = 27.861, z = 48.908). The exhaustivity parameters and the number of models were 200 and 20, respectively. After docking, each protein-ligand complex system had multiple conformations. The scores are evaluated in order of affinity, and the reasonable posture of the interaction is judged empirically. The optimal composite system is finally selected to establish the final conformation and spatial coordinates of the inhibitor as the initial conformation for the subsequent MD simulation steps [29]. PyMOL was used for graphic display.

### 2.7. Molecular Dynamics Simulation 

Based on the docking result, the ligand lies in the pocket of 1LPB chain B. The function of MD simulation is to demonstrate how the conformation of a protein-ligand complex changes during the binding process and to explain how small ligand molecules bind to receptor proteins [30].

The GROMACS package (version 19.5) is used for MD simulations of protein-ligand complexes [31]. Subsequently, 100 ns simulations were performed using AMBER14SB [32] to describe the protein field and GAFF (General AMBER Force Field) to describe the small ligand molecule. With a minimum distance of 1.0 nm between the atoms of the solute and the edge of the periodic box, the water box adopted the TIP3P water model. Overlapping water molecules were removed from the system, and the proper amounts of Na^+^/Cl^−^ ions were added to neutralize the system. After minimizing energy using the steepest descent method, the system is balanced in two steps as follows: (1) Canonical Ensemble (NVT, 0.2 ns) and (2) Isothermal-isobaric (NPT, 0.5 ns), followed by MD start of operation [30]. The default linear constraint solver algorithm was used to constrain each bond that contained a hydrogen atom. For the long-range interactions, the particle grid Evald approach was applied, with a 10 Å cutoff for the van der Waals interactions. A snapshot was taken every 1.0 ps with a time step of 2 fs [29]. Finally, the root mean square deviation (RMSD) and root mean square fluctuation (RMSF) of the complexes were analyzed using the GROMACS software package (version 19.5).

### 2.8. Combined Free Energy Calculation by MMGBSA

The binding free energy of enzyme protein receptor and ligand small molecule complexes is calculated by Molecular Mechanics/Generalized Born Surface Area (MMGBSA) [33]. This algorithm calculates the average binding free energy by extracting the architecture of a certain time interval from the MD simulation trajectory of the complex and solving complex interactions between complex molecules by decomposing and calculating the parts that constitute the binding free energy. In this study, we use a software called gmx_MMPBSA to perform MM/GBSA calculations and analyze the entire trajectory obtained from the GROMACS MD trajectory. gmx_MMPBSA’s functionality can be divided into two parts, specifically: the first stage is the preparation of the file, where the software generates the topology and trajectory in Amber format from the MD trajectory of GROMACS by paramED and determined the list of interacting residues in the energy decomposition according to a given range. In the second stage of the calculation, the software calls the sander provided by AmberTools to calculate the binding free energy using the GBSA model. The calculation of MMGBSA is widely used as a scoring function in drug design. In the present study, MMGBSA was used to obtain binding-free energies for the design of PL inhibitors.

## 3. Results and Discussion

### 3.1. QSAR Analysis

**Figure 1:**
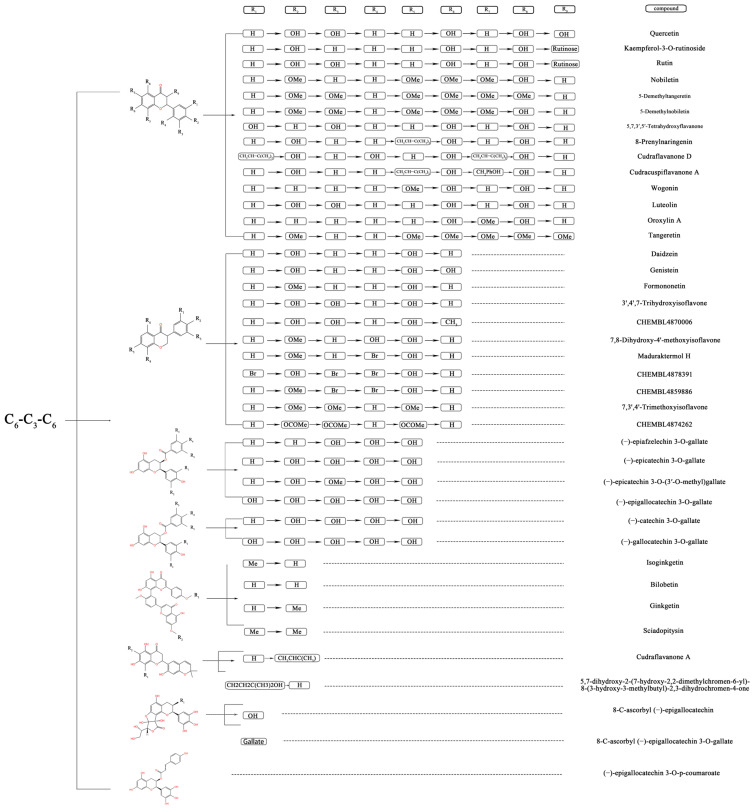
Tree structure, representing the structuring of the flavonoid compounds by the position and nature of their substituents in the aromatic ring, which were tested for their inhibitory activity against the pancreatic lipase.

To evaluate the effect of flavonoids as structural features of PL inhibitors, 40 flavonoids (Figure 1) were selected as training and testing sets to generate 2D-QSAR models (The first 29 are the training set, followed by the test set). PaDEL-Descriptor software [23] was used to generate 1444 2D descriptors to comprehensively characterize the 2D structure of flavonoids. The iterative modeling of descriptors and activity pIC_50_ values was optimized by a genetic algorithm-based MoAIcal program [18]. The methods of partial least squares and MLR were used to perform the 2D-QSAR model by establishing statistical linear correlations for five descriptors as independent variables and pIC_50_ as the dependent variable of the training data (29 compounds), resulting in the MLR-QSAR model. Based on the prediction results of the QSAR model, we were able to more accurately assess the inhibitory effect of flavonoids on PL in terms of structural features. The best model was selected on the basis of statistical parameters via the observed squared correlation coefficient (R^2^ > 0.6), which is a relative measure of the quality of fit. The cross-validated squared correlation coefficient (Q^2^) should be high as a good indicator for predicting the power of the QSAR model, and the difference between Q^2^ and R^2^ should not be more than 0.3 [25].

**Figure 2:**
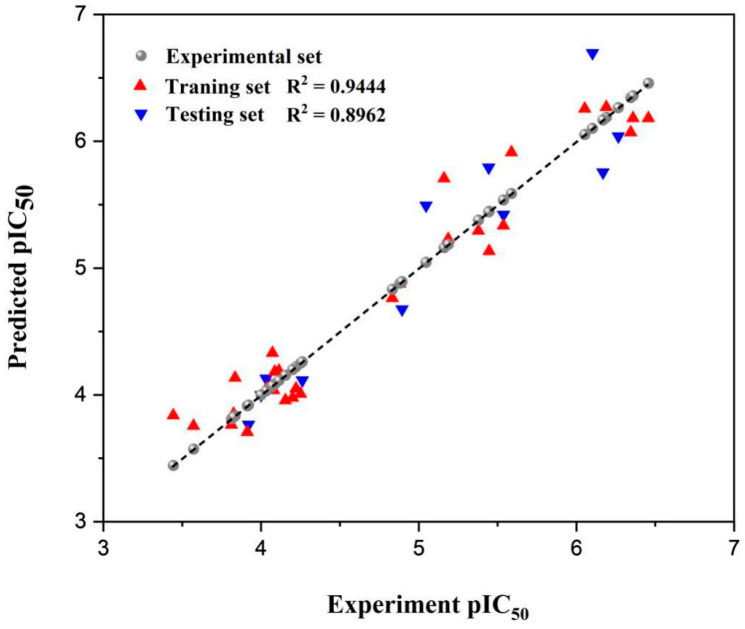
MLR-QSAR linear regression models. Predicted inhibitory activity against the experimental inhibitory activity of dataset compounds.

The QSAR model describing the inhibition of PL by flavonoids was developed, the pIC_50_ MLR equation was obtained, and results were shown in Table 1 and Figure 2. The R^2^ of the model equation was 0.9444, R^2^_adj_ = 0.9323, and the determination coefficient R^2^ of the model was larger than the adjusted R^2^, indicating that the model was not over-parameterized. The determination coefficient R^2^ (0.9444) was able to explain more than 94% of the variation in the activity values for the compounds in the data set. Figure 2 showed a good correlation between the experimental pIC_50_ and the predicted pIC_50_ based on the QSAR model.

Table 2 shows the experimental and predicted pIC_50_ values of the PL inhibition activities of 40 flavonoids. It can be seen from the residuals that the experimental values are very close to the predicted results, suggesting that the model has good predictive ability. To further evaluate the models’ predicting ability, the MAE, RSS, and SDEC parameters of the models were calculated (Table 1). The MAE of the model is 0.1754, RSS is 1.3710, and SDEC is 0.2174. A testing set was used to verify the accuracy of the model, and the R^2^, R^2^_adj_, MAE, RSS, and SDEC of the testing set were 0.8962, 0.8847, 0.2515, 1.0134, and 0.3035, respectively. These results showed that the variance between the predicted and actual values is small and the bias error is low, which further verified the prediction ability of the model.

**Figure 3:**
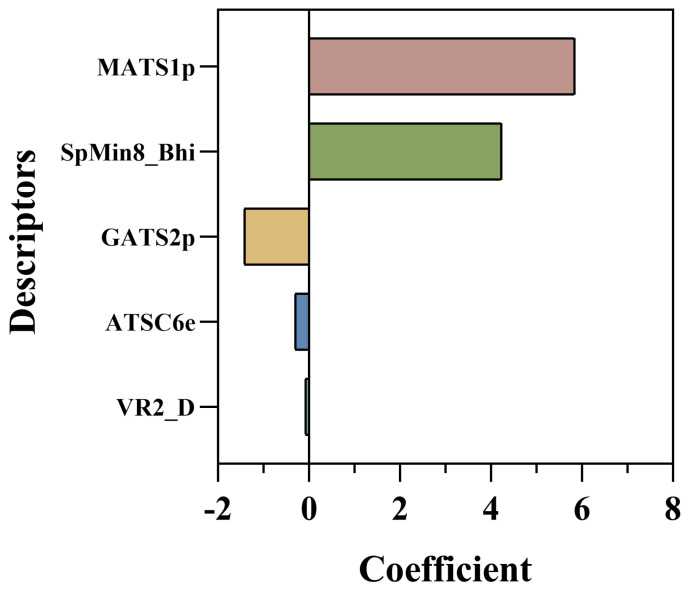
Relative importance of descriptors.

The MLR equation consists of five key descriptors: MATS1p, ATSC6e, GATS2p, SpMin8_Bhi, and VR2_D (Table 1). MATS1p has the highest coefficient of 5.84689, followed by SpMin8_Bhi of 4.23423. GATS2p, which shows that when the values of these three descriptors are larger, their inhibition of PL is greater. ATSC6e and VR2_D have negative coefficients of −1.41739, −0.30375, and −0.07596, indicating that the compounds can only inhibit PL if these descriptors have negative values. To further analyze the relationship between the structural properties of the flavonoids and the pIC_50_ values, we analyzed the importance of the descriptors in the model according to the percentage of coefficients (Figure 3). MATS1p corresponds to the Moran autocorrelation function/weighted polarization rate, which is used to determine whether there is autocorrelation in the space. The MATS1p value will be distributed between [−1, 1], with the range of [0, 1] indicating a positive correlation in the degree of aggregation among the structures and between [−1, 0] indicating a negative correlation. Since the coefficient of MATS1p in the equation is 5.84689, the different values of MATS1p will have a greater contribution to the model prediction, and the higher the absolute value of the values in the range of [0, 1] as well as [−1, 0], the more positive/negative contribution to the model and consequently the more potential for PL inhibition [34]. For example, the compounds predicted to have superior inhibition (CNP0186639, CNP0221970, and CNP0358253) had higher MATS1p and were all 0.244810577 (Table S1), suggesting a positive correlation in the degree of aggregation between their structures and thus a better effect on PL inhibition. ATSC6e corresponds to the centered Broto-Moreau autocorrelation, weighted by the Sanderson electronegativity. Similarly, the ATSC6e numerical distribution is in both the positive and negative domains and therefore contributes differently to the model depending on the positive and negative domains. SpMin8_Bhi is the minimum absolute eigenvalue of the Burden correction matrix—n8/weighted by the relative first ionization potential [26]. The coefficient of SpMin8_Bhi has positive values, and since the value distributions of the compounds are positive, SpMin8_Bhi is positively correlated to the predictive power of the model. GATS2p is the molecular descriptor of Geary autocorrelation—lag2/weighted by polarization rate [34]. VR2_D indicates the normalized Landecker eigenvector index based on the topological distance matrix to describe the topological distance [35]. In contrast, the distribution of the values of GATS2p and VR2_D were both positive, but due to their negative coefficients, GATS2p and VR2_D values are negatively correlated with the model prediction results, while VR2_D’s contribution is small.

**Figure 4:**
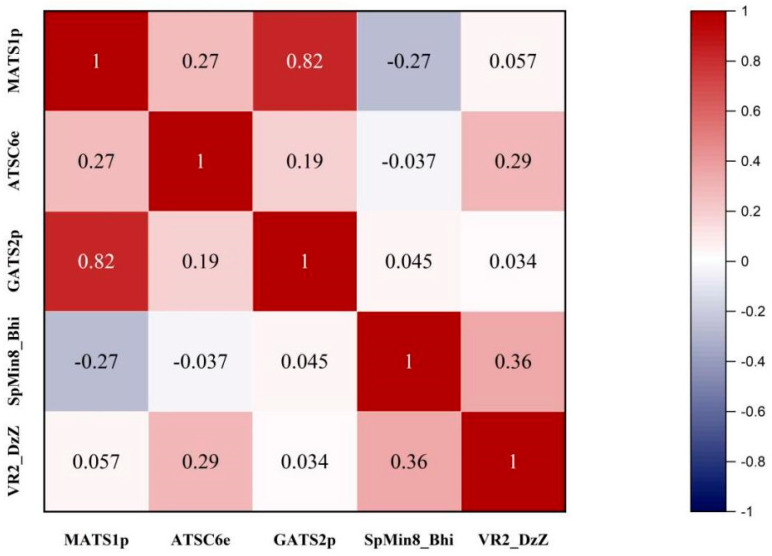
Optimal autocorrelation analysis in MLR−QSAR models.

The model descriptor correlation matrix illustrated in Figure 4 based on Pearson’s correlation coefficients showed that the inter-correlation between most of the two descriptors of the model is less than 0.5, indicating that the descriptors are orthogonal to each other and there is no multicollinearity problem in the model. Therefore, the constructed model can be used to quantify the inhibitory activity of natural flavonoids on PL.

### 3.2. Discovery of Natural PL Inhibitors and ADMET Analysis

QSAR models were developed to predict the inhibitory activity of compounds in COCONUT (https://coconut.naturalproducts.net, accessed on 10 December 2022), a natural product library. The MolNatSim program, developed based on molecular smiles and clustering algorithms, was used to scan all molecules in the COCONUT database. 1061 clustered molecules matching 40 flavonoids were retrieved. The pIC_50_ values of the preliminary screened flavonoids were subsequently predicted by the QSAR model, and the results were ranked in descending order. The top five substances in the pIC_50_ prediction results were screened for analysis (Table S2). ADMET prediction and manual examination were carried out, respectively. Of the 5 ligands considered in this study, compounds [4-[5,7-dihydroxy-3-(3,4,5-trihydroxybenzoyl)oxy-3,4-dihydro-2H-chromen-2-yl]-2,6-dihydroxyphenyl]2,3,4-trihydroxybenzoate (CNP0186639), Epigallocatechin 3,3′,-Di-*O*-Gallate (CNP0221970), [5-[5,7-dihydroxy-3-(3,4,5-trihydroxybenzoyl)oxy-3,4-dihydro-2H-chromen-2-yl]-2,3-dihydroxyphenyl]2,3,4-trihydroxybenzoate (CNP0358253), Isoorientin 2″-*O*-Gallate (CNP0286940), and 3-*O*-Galloylmucic acid (CNP0206087) (Table 3) were selected due to their relatively lower IC_50pre_ of 0.49, 0.61, 0.73, 0.85, and 1.27 μM, respectively.

PreADME was used to analyze the ADMET processes of the target compounds after intake and to assess bioavailability, and the results are shown in Table 3. All five natural products were moderately soluble, which would facilitate GI absorption and blood distribution. However, the bioavailability score and GI absorption predictions for all five target compounds were low, so future consideration could be given to changing the administration mode or selecting a suitable carrier administration to improve their absorption and utilization. Pgp is a transporter protein responsible for the excretion of harmful substances, and the results showed that none of the natural products were substrates of Pgp, suggesting that selected compounds will not be metabolized by Pgp and excreted very quickly. Toxicological results showed that only CNP0286940 was not expected to exhibit carcinogenicity in rats, while all target compounds did not exhibit carcinogenicity in mice. The percutaneous permeability coefficient log Kp (cm/s) results can further evaluate the potential of the target compound as a topical dressing. The analysis of the above results provides complementary data support for our model to predict the outcome of natural PL inhibitors. 

In particular, both CNP0186639 and CNP0358253 carry the structure of gallic acid on the B ring, and the only difference between them is the position of the gallic acid on the C ring, whereas CNP0186639 has a strong inhibitory effect on PL with an IC_50pre_ value of 0.49 μM. In other studies, gallic acid has shown significant PL inhibition and reduced obesity [17]. Considering that groups such as gallic acid are more hydrophobic, this suggests that these groups may enhance the inhibitory effect of the compounds on PL by increasing the hydrophobic binding capacity.

### 3.3. Molecular Docking Analysis

Molecular docking is a process of mutual recognition between receptor and ligand molecules. Molecular docking was performed in this study to verify the rationality of the pharmacological model of PL 2D-QSAR. AutoDock-Vina was used for molecular docking, and the best conformation was selected for each compound. The binding energy of all target compounds was lower than that of the positive control, Orlistat, with −6.7 kcal mol^−1^ (Table 4). Results showed that the compounds predicted by the model were well bound with PL, indicating the accuracy of the model. The lowest binding energy of CNP0186639 was −9.6 kcal mol^−1^, while the binding energies of the other compounds ranged from −8.4 to −9.5 kcal mol^−1^, which was lower than that of the positive control. Meanwhile, CNP0186639 was the compound with the lowest pIC_50pre_ in the QSAR predicted results, indicating that it can be recommended as a potential PL inhibitor.

**Figure 5:**
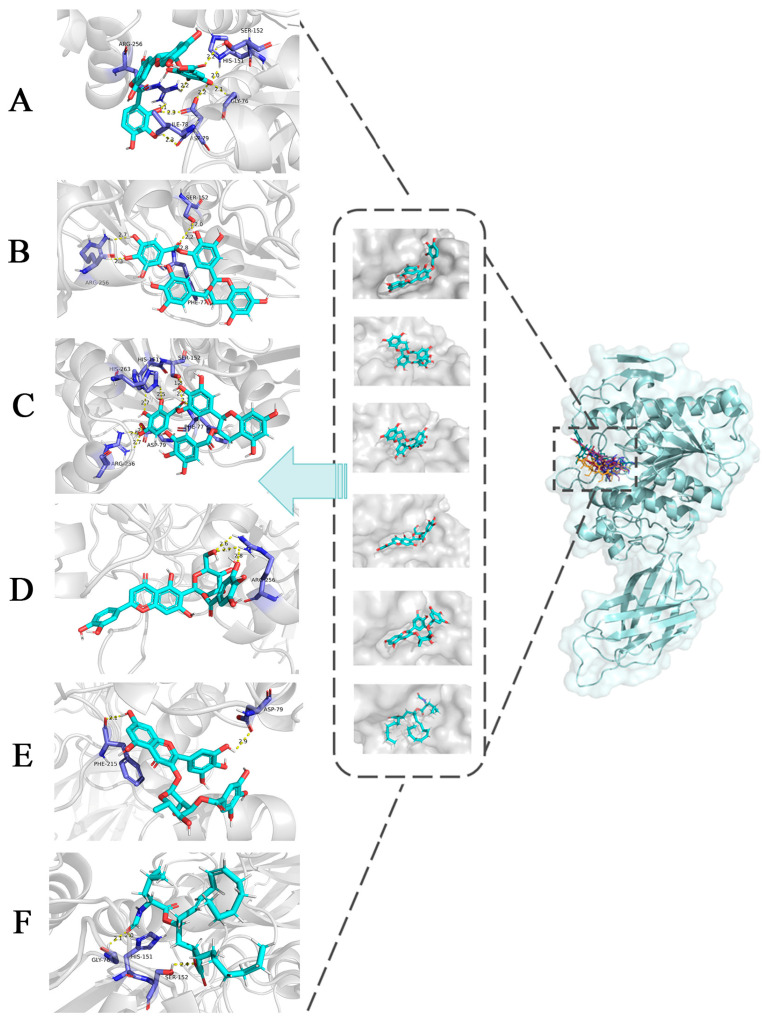
Docking diagrams of the screened compounds with the pancreatic lipase enzyme molecule. Hydrogen bonds are represented by yellow dotted lines. (**A**) PL-CNP0186639. (**B**) PL-CNP0221970. (**C**) PL-CNP0358253. (**D**) PL-CNP0286940. (**E**) PL-CNP0206087. (**F**) Orlistat (Positive control).

Visual analysis of the docking results was carried out with pymol, and the results showed that there were many interactions between the screened natural compounds and 1LPB. In Figure 5, the target compounds were tightly bound to PL (1LPB), and the yellow dashed lines represented the hydrogen bond formed by the binding of the receptor to the ligand. It can be observed from the docking surface figure (Figure 5) that the ligand is well embedded in the hydrophobic pocket of the receptor. CNP0186639 formed a maximum of eight hydrogen bonds with Ser152, His151, Gly76, Asp79, Ile78, and Arg256 of the acceptor, which was the highest number of hydrogen bonds among the target compounds. CNP0221970, CNP0358253, CNP0286940, and CNP0206087 formed five, eight, three, and two hydrogen bonds with the protein receptor, respectively, and the inhibitory properties of the substances to PL decreased with the reduction of the number of hydrogen bonds, except for CNP0358253. This may be due to the fact that the structures of CNP0358253 and CNP0186639 are very similar, both of which have gallic acid attached at position three of the B ring, and the difference is that the linking sites of the connecting groups on the C ring are inconsistent. CNP0221970 interacted with the active site of PL and formed hydrogen bonds with Phe77, Ser152, and Arg256. CNP0358253 showed similar amino acid residue interactions as CNP0221970, with Asp79, His151, and His263 also interacting to form hydrogen bonds besides Phe77, Ser152, and Arg256. CNP0286940 bound to Arg256 of the active pocket to form hydrogen bonds, while CNP0206087 formed hydrogen bonding interactions with Asp79 and Phe215. Arg256, Ser152, and Phe77 were assumed to be the key residues that determine the stability and low docking energy of the docking complex. As seen in Figure 5F, the control group (orlistat) formed hydrogen bonds with Gly76, His151, and Ser152 amino acid residues of the protein, which were also action sites of the target compound, further verifying the feasibility of binding the target compound to the receptor.

It has been suggested that hydrogen bonding interactions play key roles in stabilizing enzyme-ligand complex catalysis and depend on the number of hydrogen bonds [36]. All five natural compounds screened by the QSAR model in this study could form hydrogen bonds with multiple residues of PL, and the number of hydrogen bonds formed was greater than the positive control. These results confirmed the potential of the target compounds as inhibitors of PL and the accuracy of the QSAR model.

### 3.4. Molecular Dynamics Simulation Analysis

MD is generally used in conjunction with molecular docking to further explore the mechanisms of ligand-protein interactions [37]. MD simulations of the target compounds were performed using the GROMACS 2019 software package and analyzed using different trajectories. The RMSD trajectory curves can determine the average deviation between the complex conformation and the original conformation over a certain time period and evaluate whether the system has reached a steady state [38].

**Figure 6:**
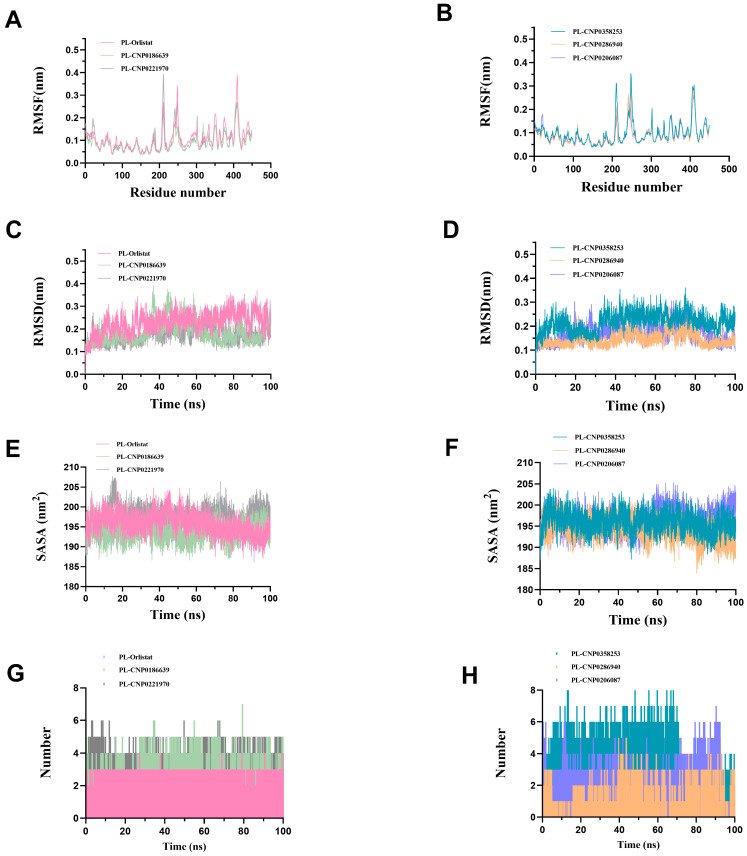
Molecular dynamics (100 ns) results for six enzymatic. (**A**) Root mean square fluctuation (RMSF, nm) of PL-Orlistat, PL-CNP0186639, and PL-CNP0221970. (**B**) Root mean square fluctuation (RMSF, nm) of PL-CNP0358253, PL-CNP0286940, and PL-CNP0206087. (**C**) Root mean square deviations (RMSD, nm) of PL-Orlistat, PL-CNP0186639, and PL-CNP0221970. (**D**) Root mean square deviations (RMSD, nm) of PL-CNP0358253, PL-CNP0286940, and PL-CNP0206087. (**E**) Solvent Accessible Surface of PL-Orlistat, PL-CNP0186639, and PL-CNP0221970. (**F**) Solvent Accessible Surface of PL-CNP0358253, PL-CNP0286940, and PL-CNP0206087. (**G**) The number of hydrogen bonds formed between the active compounds and 1LPB of PL-Orlistat, PL-CNP0186639, and PL-CNP0221970. (**H**) The number of hydrogen bonds formed between the active compounds and 1LPB of PL-CNP0358253, PL-CNP0286940, and PL-CNP0206087.

The RMSF curves were used to study the partial variations of the protein chain residues. Figure 6A,B showed the fluctuation curves of the amino acid residues of the complexes. The values of all five complex systems were below 0.4 nm, and stable fluctuations occurred around 0.1 nm, which provided a suitable basis for subsequent analysis. Binding of five natural compounds to the PL receptor resulted in increased flexibility of residues in the key regions (200–225), (230–260), and (400–420), suggesting that the novel natural products may inhibit PL activity by interacting with key residues that affect the active pocket. Among these complex systems of compounds binding to PL, PL-CNP0186639 showed the smallest overall fluctuation, with a peak of 0.27 nm at residue 410. The fluctuations of PL-CNP0206087 were also slight, again peaking at 0.27 nm near 410 amino acid residues. PL-CNP0286940 showed an enhanced fluctuation, peaking at 0.32 nm around 250 nm. The peaks of PL-Orlistat and PL-CNP018663 were close to 0.39 nm, and the peaks of PL-CNP0358253 were slightly above 0.35 nm, all with large fluctuations. In conclusion, the fluctuating trajectories of the target compound-receptor binding complexes followed the same pattern as that of PL-Orlistat, indicating that the five screened potential inhibitors showed low conformational changes and high stability upon binding to PL.

As shown in the Figure 6C,D, the PL-Orlistat system reached a stable state at 30 ns, and the RMSD value was stable at 0.25 nm. The PL-CNP0186639 and PL-CNP0221970 systems reached a balance near 40 ns, and their RMSD values were stable at about 0.17 nm, while the RMSD values of the PL-CNP0196639 system showed a certain decrease after 60 ns. The PL-CNP0358253 system reached stability at around 30 ns and maintained it at around 0.23 nm. Meanwhile, the PL-CNP0286940 and PL-CNP0206087 systems reached equilibrium faster within 20 ns and stabilized at 0.13 nm and 0.16 nm, respectively. The overall fluctuations of the PL-CNP022197, PL-CNP0286940, and PL-CNP0206087 complexes were small, even within a smaller fluctuation range than the control system. In addition, the fluctuations in the RMSD analyses of protein and small molecule complex systems, and small molecules alone were all within 0.2 nm, and no significant fluctuations in conformational changes were observed (Figure S1). In conclusion, In summary, small molecules and receptors form conformational systems and their respective relative stability and equilibrium over 100 ns simulation time, suggesting that the 100 ns trajectory conformations of the proteins do not show substantial structural differences, implying that the ligands and complexes are structurally stable.

Solvent Accessible Surface Area (SASA) calculates the area over which a solute can interact with a solvent molecule through van der Waals forces. The SASA value of a protein decreases as protein densities increase, so changes in SASA can predict changes in protein structure. The binding of any small molecule may change the SASA value, sometimes dramatically affecting the structure of the protein. Figure 6E,F shows the SASA changes of the screened potential PL inhibitor and receptor systems during 100 ns MD simulations. It can be seen that PL-CNP0221970 has a peak near 17 ns and then declines to level off, suggesting that the receptor opens the hydrophobic pocket first and becomes more compact in structure following binding to small molecules. Similarly, the SASA results for the PL-orlistat system show a decreasing trend in the latter part, suggesting that the binding of orlistat to PL makes its structure more compact. The SASA of other small molecules and receptor-bound systems was mainly between 185–205 nm^2^. PL-CNP0286940 had the lowest peak (183.7 nm^2^) at 80 ns, indicating a very tight binding in PL to CNP0286940, which could be potentially related to the glycoside of CNP0286940. CNP0206087 had the relatively lowest inhibitory performance among the potential PL inhibitors screened, and the latter portion of its SASA was higher compared to the others, suggesting poorer binding to PL compared to the other compounds.

In addition, we analyzed the number of hydrogen bonds for trajectories lasting 100 ns. Figure 6G,H shows the hydrogen bonding interactions between the small molecule ligand and 1LPB at a distance of 3.5 Å. The maximum number of hydrogen bonds formed by Orlistat, CNP0186639, CNP0221970, CNP0358253, CNP0286940, and CNP0206087 with 1LPB is 4, 7, 6, 8, 5, and 7, respectively. During 100 ns, 8 hydrogen bonds were formed between CNP0358253 and 1LPB, which is the highest number of hydrogen bonds formed during the whole simulation, ensuring better stability. Moreover, the potential inhibitors screened by the model were able to form more hydrogen bonds with 1LPB and all of them were more than Orlistat, suggesting that their hydrogen bonding interactions with the protein receptor are more abundant and have better stability than Orlistat, which also explains their superior binding activity. 

### 3.5. Combining Free Energy Calculations 

In this study, in order to adequately analyze the potential interaction of the target residues with the ligand substructure and to quantitatively describe the interaction mechanism between the five potential natural products and PL, energy calculations were performed based on the trajectories obtained from the MD results. After obtaining conformations at 0.1 ns intervals in the 100 ns dynamics trajectory, energy calculations were performed for the complex system using the MMGBSA algorithm based on Poisson-Boltzmann energy theory. The results for the different energy contributions of several compounds are shown in Table 5. ΔEvdw, ∆Eele, and ∆Gnon-pol were negative, indicating that van der Waals forces, electrostatic interactions, and nonpolar solvation energy contribute positively to the bonding, and that van der Waals forces and electrostatic interactions perform a major role. The ∆Ggas is obtained by summing the bonding component (bonding force + angular force + dihedral angular force) and the non-bonding component (van der Waals force + electrostatic interaction) [39]. ∆gpol is the polar solvation energy and exhibits a positive energy contribution that is detrimental to the stability of the system, inhibiting the spontaneous binding of the natural inhibitor to PL. The negative binding free energy indicates that the binding of ligands and receptors proceeds spontaneously and can reach a steady state, and the lower the energy, the more stable the system is. In the results of MMGBSA energy calculations, all compounds showed good binding free energies, with simulated values of −35.68, −33.22, −32.3, −44.35, −30.77, and −22.89 kcal/mol for the five target compounds, respectively. It can be predicted that CNP0358253, CNP0186639, and CNP0221970 can bind to PL to form more stable complexes and therefore have more potential to become natural inhibitors of PL.

## 4. Conclusions

In this study, a predictable anti-PL natural product QSAR model were developed using the artificial intelligence drug design software MolAIcal based on genetic algorithms and their conformational relationships. Five potentially efficient anti-PL natural products were screened based on this novel QSAR model and ADMET. Subsequently, molecular docking, MD simulation, and MGBSA free energy calculation were performed to not only reveal the interaction of candidate novel natural products with the amino acid residues of PL but also to validate the stability of these novel natural compounds bound to PL. In all, [4-[5,7-dihydroxy-3-(3,4,5-trihydroxybenzoyl)oxy-3,4-dihydro-2H-chromen-2-yl]-2,6-dihydroxyphenyl]2,3,4-trihydroxybenzoate (CNP0186639), Epigallocatechin 3,3′,-Di-*O*-Gallate (CNP0221970), [5-[5,7-dihydroxy-3-(3,4,5-trihydroxybenzoyl)oxy-3,4-dihydro-2H-chromen-2-yl]-2,3-dihydroxyphenyl]2,3,4-trihydroxybenzoate (CNP0358253), Isoorientin 2″-*O*-Gallate (CNP0286940), and 3-*O*-Galloylmucic acid (CNP0206087) can be considered as potential novel PL inhibitors.

## Figures and Tables

**Table 1:** QSAR model parameters and cross-validation results.

Train		Test	
R^2^	0.9444	R^2^	0.8962
R^2^_adj_	0.9323	R^2^_adj_	0.8847
MAE	0.1754	MAE	0.2515
RSS	1.3710	RSS	1.0134
SDEC	0.2174	SDEC	0.3035
pIC_50pre_ = 3.83259 + (5.84689) × MATS1p + (−0.30375) × ATSC6e + (−1.41739) × GATS2p + (4.23423) × SpMin8_Bhi + (−0.07596) × VR2_D			

**Table 2:** Experimental and predicted pIC_50_ values of pancreatic lipase inhibition activities of 40 flavonoids (The training set has 29 and the test set has 11).

Flavonoid	pIC_50_ a	pIC_50pre_ b	Residual
Daidzein	4.081	4.037	0.044
Genistein	4.222	4.050	0.172
3′,4′,7-Trihydroxyisoflavone	4.155	3.957	0.198
CHEMBL4870006	4.036	4.051	0.015
Maduraktermol H	4.201	3.977	0.224
CHEMBL4878391	4.060	4.072	0.011
7,3′,4′-Trimethoxyisoflavone	3.444	3.838	0.394
CHEMBL4874262	3.914	3.707	0.207
Wogonin	3.813	3.765	0.049
Oroxylin A	4.251	4.007	0.244
(−)-Epiafzelechin 3-*O*-gallate	5.588	5.912	0.323
(−)-Epicatechin 3-*O*-gallate	6.345	6.070	0.275
(−)-Epigallocatechin 3-*O*-gallate	6.457	6.181	0.276
(−)-Epigallocatechin 3-*O*-*p*-coumaroate	6.053	6.256	0.203
(−)-Gallocatechin 3-*O*-gallate	6.360	6.181	0.178
8-C-ascorbyl (−)-epigallocatechin	6.190	6.268	0.079
Tangeretin	4.833	4.763	0.070
Nobiletin	4.880	4.876	0.004
5-Demethylnobiletin	5.379	5.294	0.085
Quercetin	3.836	4.134	0.298
Bilobetin	5.447	5.133	0.315
Ginkgetin	5.161	5.706	0.545
5,7,3′,5′-Tetrahydroxyflavanone	4.087	4.182	0.094
8-Prenylnaringenin	4.114	4.190	0.076
Cudraflavanone A	5.187	5.229	0.041
2′,5,7-Trihydroxy-4,5′-(2,2-dimethylchromeno)-8-(3-hydroxy-3-methylbuthyl)flavanone	4.073	4.330	0.257
Luteolin	3.573	3.755	0.182
Kaempferol-3-Orutinoside	5.538	5.335	0.203
Rutin	3.827	3.850	0.023
Formononetin *	3.921	3.765	0.156
7,8-Dihydroxy-4′-methoxyisoflavone *	4.032	4.128	0.097
CHEMBL4859886 *	4.000	3.998	0.002
(−)-Epicatechin 3-O-(3′-*O*-methyl)gallate *	6.167	5.755	0.413
(−)-Catechin 3-*O*-gallate *	6.265	6.039	0.226
8-C-ascorbyl (−)-epigallocatechin 3-*O*-gallate *	6.102	6.696	0.594
5-Demethyltangeretin *	5.444	5.793	0.349
Isoginkgetin *	5.538	5.421	0.117
Sciadopitysin *	4.893	4.674	0.219
Cudraflavanone D *	5.046	5.493	0.448
Cudracuspiflavanone A *	4.261	4.115	0.146

a. pIC_50_ means experimental value. b. pIC_50pre_ means predicted pIC_50_ values. * test set.

**Table 3:** QSAR prediction and ADMET analysis of potential natural pancreatic lipase inhibitors.

Compounds	Molecular Structure	IC_50pre_ (μM)	Water Solubility	BS	GI Absorption	Pgp Substrate	log Kp (cm/s)	Carcino_Mouse	Carcino_Rat
CNP0186639	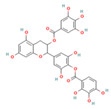	0.49	Moderately soluble	0.17	Low	No	−7.55	negative	positive
CNP0221970	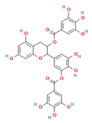	0.61	Moderately soluble	0.17	Low	No	−7.94	negative	positive
CNP0358253	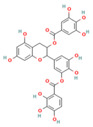	0.73	Moderately soluble	0.17	Low	No	−7.55	negative	positive
CNP0286940	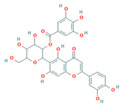	0.85	Moderately soluble	0.17	Low	No	−9.24	negative	negative
CNP0206087	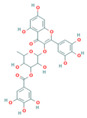	1.27	Moderately soluble	0.17	Low	No	−8.87	negative	positive

Note: IC_50pre_ means predicted IC_50_ values; BS means Bioavailability Score. CNP0186639, [4-[5,7-dihydroxy-3-(3,4,5-trihydroxybenzoyl)oxy-3,4-dihydro-2H-chromen-2-yl]-2,6-dihydroxyphenyl]2,3,4-trihydroxybenzoate; CNP0221970, Epigallocatechin 3,3′,-Di-O-Gallate; CNP0358253, [5-[5,7-dihydroxy-3-(3,4,5-trihydroxybenzoyl)oxy-3,4-dihydro-2H-chromen-2-yl]-2,3-dihydroxyphenyl]2,3,4-trihydroxybenzoate; CNP0286940, Isoorientin 2″-O-Gallate; CNP0206087, 3-O-Galloylmucic acid.

**Table 4:** The affinity of compounds with pancreatic lipase (1LPB) (kcal mol^−1^).

Active Compound	Protein (PDBID)	Docking Energy (kcal/mol)
Orlistat	pancreatic lipase (1LPB)	−6.7
CNP0186639	pancreatic lipase (1LPB)	−9.6
CNP0221970	pancreatic lipase (1LPB)	−9.2
CNP0358253	pancreatic lipase (1LPB)	−9.5
CNP0286940	pancreatic lipase (1LPB)	−9.3
CNP0206087	pancreatic lipase (1LPB)	−8.4

Note: CNP0186639, [4-[5,7-dihydroxy-3-(3,4,5-trihydroxybenzoyl)oxy-3,4-dihydro-2H-chromen-2-yl]-2,6-dihydroxyphenyl]2,3,4-trihydroxybenzoate; CNP0221970, Epigallocatechin 3,3′,-Di-O-Gallate; CNP0358253, [5-[5,7-dihydroxy-3-(3,4,5-trihydroxybenzoyl) oxy-3,4-dihydro-2H-chromen-2-yl]-2,3-dihydroxyphenyl]2,3,4-trihydroxybenzoate; CNP0286940, Isoorientin 2″-O-Gallate; CNP0206087, 3-O-Galloylmucic acid.

**Table 5:** Binding free energy of complex formation between pancreatic lipase and its inhibitors.

Energy (kJ/mol)	ΔEvdw	∆Eele	∆Gpol	∆Gnon-pol	∆Ggas	∆Gsol	∆Gbind
Orlistat	−37.56	−33.32	40.95	−5.75	−70.88	35.2	−35.68
CNP0186639	−42.87	−31.17	46.74	−5.92	−74.04	40.82	−33.22
CNP0221970	−36.33	−53.92	63.46	−5.51	−90.26	57.96	−32.3
CNP0358253	−36.66	−71.72	70.01	−5.99	−108.38	64.03	−44.35
CNP0286940	−45.42	−19.41	39.72	−5.67	−64.83	34.06	−30.77
CNP0206087	−38.78	−40.53	61.83	−5.41	−79.31	56.42	−22.89

Note: ΔEvdw, the van der Waals interaction energy term; ∆Eele, the electrostatic interaction energy term; ∆Gpol, the polar solvation energy term; ∆Gnon-pol, The non-polar solvation energy term; ∆Ggas, the gas phase free energy term; ∆Gsol, the solvation free energy term; ∆Gbind, the free energy of binding. CNP0186639, [4-[5,7-dihydroxy-3-(3,4,5-trihydroxybenzoyl) oxy-3,4-dihydro-2H-chromen-2-yl]-2,6-dihydroxyphenyl]2,3,4-trihydroxybenzoate; CNP0221970, Epigallocatechin 3,3′,-Di-O-Gallate; CNP0358253, [5-[5,7-dihydroxy-3-(3,4,5-trihydroxybenzoyl)oxy-3,4-dihydro-2H-chromen-2-yl]-2,3-dihydroxyphenyl]2,3,4-trihydroxybenzoate; CNP0286940, Isoorientin 2″-O-Gallate; CNP0206087, 3-O-Galloylmucic acid.

## Data Availability

Data from other references cited in this study were collated and added to the Appendix, and the rest of the model data are original and have not been improperly selected, manipulated, enhanced, or fabricated.

## Supplementary Materials

The following supporting information can be downloaded at: https://www.mdpi.com/article/10.3390/nu15153489/s1, Figure S1: RMSD analysis of complex (A) and ligand (B), Table S1: Reference sources for each compound in the dataset, Table S2: Molecular descriptors of potential PL inhibitors screened [40,41,42,43].
