# Supplementary material for: Construction of a QSAR Model Based on Flavonoids and Screening of Natural Pancreatic Lipase Inhibitors
Source: Nutrients. 2023 Aug 7;15(15):3489. doi: 10.3390/nu15153489 (PMC10421515; doi:10.3390/nu15153489)
Supplement: Supplementary file 1 [file nutrients-15-03489-s001.zip › nutrients-2504911-supplementary.pdf]

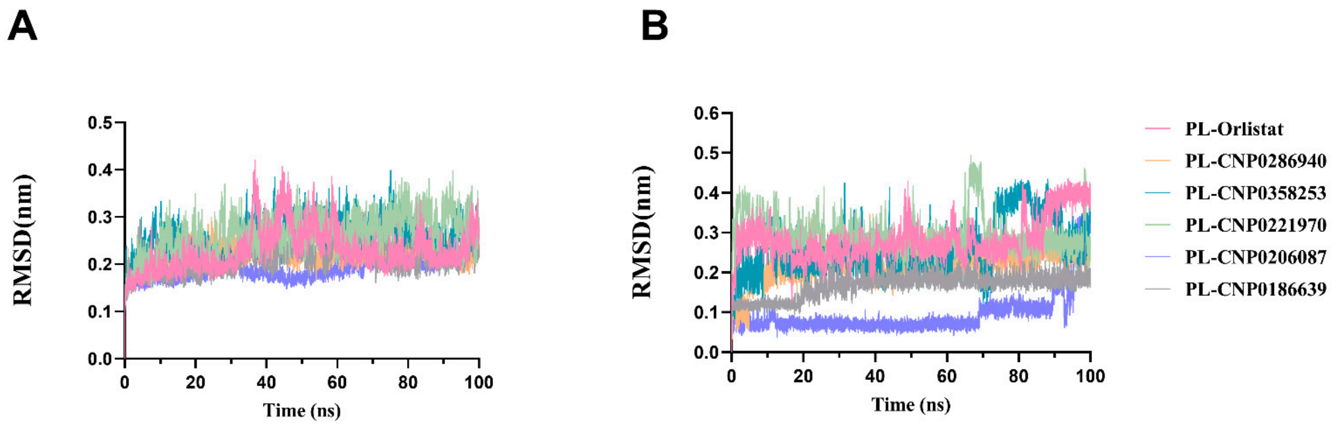

Figure S1. RMSD analysis of complex (A) and ligand (B) of PL-Orlistat, PL-CNP0286940, PL-CNP0358253, PL-CNP0221970, PL-CNP0206087 and PL-CNP0186639.

Table S1. Reference sources for each compound in the dataset.

Number	Name of compound	References
1	Daidzein	
2	genistein	
3	formononetin	
4	3',4',7-Trihydroxyisoflavone	
5	CHEMBL4870006	[1]
6	7,8-Dihydroxy-4'-methoxyisoflavone	
7	Maduraktermol H	
8	CHEMBL4878391	
9	CHEMBL4859886	
10	7,3',4'-Trimethoxyisoflavone	
11	wogonin	[2]

12	oroxylin A	
13	(−)-epiafzelechin 3-O-gallate	
14	(−)-epicatechin 3-O-gallate	
15	(−)-epicatechin 3-O-(3′-O-methyl)gallate	
16	(−)-epigallocatechin 3,5-di-O-gallate	
17	(−)-epigallocatechin 3-O-p-coumaroate	
18	(−)-epigallocatechin 3-O-gallate	[3]
19	(−)-catechin 3-O-gallate	
20	(−)-catechin 3-O-gallate	
21	8-C-ascorbyl (−)-epigallocatechin	
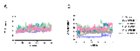 22	8-C-ascorbyl (−)-epigallocatechin 3-O-gallate	
23	tangeretin	
24	nobiletin	[4]
25	5-demethyltangeretin	
26	5-demethylnobiletin	
27	Isoginkgetin	
28	Bilobetin	[5]
29	Ginkgetin	
30	Sciadopitysin	
31	5,7,3′,5′-tetrahydroxyflavanone	
32	8-prenylnaringenin	[6]
33	cudraflavanone D	

34	cudraflavanone A	
35	2′,5,7-trihydroxy-4,5′-(2,2-dimethylchromeno)-8-(3-hydroxy-3-methylbuthyl)flavanone	
36	cudracuspiflavanone A	
37	Luteolin	[7]
38	Kaempferol-3-Orutinoside	
39	Rutin	
40	Quercetin	

Reference:

1. Cardullo, N.; Muccilli, V.; Pulvirenti, L.; Tringali, C. Natural Isoflavones and Semisynthetic Derivatives as Pancreatic Lipase Inhibitors. *J. Nat. Prod.* **2021**, *84*, 654–665, doi:10.1021/acs.jnatprod.0c01387.
2. Wan, L.-H.; Jiang, X.-L.; Liu, Y.-M.; Hu, J.-J.; Liang, J.; Liao, X. Screening of Lipase Inhibitors from Scutellaria Baicalensis Extract Using Lipase Immobilized on Magnetic Nanoparticles and Study on the Inhibitory Mechanism. *Anal Bioanal Chem* **2016**, *408*, 2275–2283, doi:10.1007/s00216-016-9320-7.
3. Nakai, M.; Fukui, Y.; Asami, S.; Toyoda-Ono, Y.; Iwashita, T.; Shibata, H.; Mitsunaga, T.; Hashimoto, F.; Kiso, Y. Inhibitory Effects of Oolong Tea Polyphenols on Pancreatic Lipase in Vitro. *J. Agric. Food Chem.* **2005**, *53*, 4593–4598, doi:10.1021/jf047814+.
4. Huang, X.; Zhu, J.; Wang, L.; Jing, H.; Ma, C.; Kou, X.; Wang, H. Inhibitory Mechanisms and Interaction of Tangeretin, 5-Demethyltangeretin, Nobiletin, and 5-Demethylnobiletin from Citrus Peels on Pancreatic Lipase: Kinetics, Spectroscopies, and Molecular Dynamics Simulation. *International Journal of Biological Macromolecules* **2020**, *164*, 1927–1938, doi:10.1016/j.ijbiomac.2020.07.305.
5. Liu, P.-K.; Weng, Z.-M.; Ge, G.-B.; Li, H.-L.; Ding, L.-L.; Dai, Z.-R.; Hou, X.-D.; Leng, Y.-H.; Yu, Y.; Hou, J. Biflavones from Ginkgo Biloba as Novel Pancreatic Lipase Inhibitors: Inhibition Potentials and Mechanism. *International Journal of Biological Macromolecules* **2018**, *118*, 2216–2223, doi:10.1016/j.ijbiomac.2018.07.085.
6. Jo, Y.H.; Kim, S.B.; Liu, Q.; Lee, J.W.; Hwang, B.Y.; Lee, M.K. Benzylated and Prenylated Flavonoids from the Root Barks of Cudrania Tricuspidata with Pancreatic Lipase Inhibitory Activity. *Bioorganic & Medicinal Chemistry Letters* **2015**, *25*, 3455–3457, doi:10.1016/j.bmcl.2015.07.017.
7. Habtemariam, S. Antihyperlipidemic Components of *Cassia Auriculata* Aerial Parts: Identification Through *In Vitro* Studies: ANTIHYPERLIPIDEMIC COMPONENTS OF *CASSIA AURICULATA* AERIAL PARTS. *Phytother. Res.* **2013**, *27*, 152–155, doi:10.1002/ptr.4711.

Table S2. Molecular descriptors of potential PL inhibitors screened.

Name	MATS1p	ATSC6e	GATS2p	SpMin8_Bhi	VR2_DzZ
CNP0186639	0.244810577	0.796184389	1.176948987	0.947291746	13.86796247
CNP0221970	0.244810577	1.661881994	1.176948987	0.968044715	12.85192419
CNP0358253	0.244810577	2.010529781	1.176948987	0.96789442	12.44882367
CNP0286940	0.233223414	1.584163789	1.177845666	0.950973695	13.17518648
CNP0206087	0.199429013	1.535463529	1.182106191	0.953684305	13.15952316
